# Gut Microbiome Alterations During HIV/SIV Infection: Implications for HIV Cure

**DOI:** 10.3389/fmicb.2019.01104

**Published:** 2019-05-22

**Authors:** Katti R. Crakes, Guochun Jiang

**Affiliations:** ^1^Department of Medical Microbiology and Immunology, University of California, Davis, Davis, CA, United States; ^2^Department of Biochemistry and Biophysics, Institute for Global Health & Infectious Diseases, UNC HIV Cure Center, The University of North Carolina at Chapel Hill, Chapel Hill, NC, United States

**Keywords:** human immunodeficiency virus, simian immunodeficiency virus, gut microbiome, mucosal immunity, antiretroviral (ARV) therapy

## Abstract

Gut mucosal damage, associated with Human Immunodeficiency Virus-1 (HIV) infection, is characterized by depletion in CD4^+^ T cells and persistent immune activation as a result of early epithelial barrier disruption and systemic translocation of microbial products. Unique approaches in studying both HIV infection in human patients and Simian Immunodeficiency Virus (SIV) infection in rhesus macaques have provided critical evidence for the pathogenesis and treatment of HIV/AIDS. While there is vast resemblance between SIV and HIV infection, the development of gut dysbiosis attributed to HIV infection in chronically infected patients has not been consistently reported in SIV infection in the non-human primate model of AIDS, raising concerns for the translatability of gut microbiome studies in rhesus macaques. This review outlines our current understanding of gut microbial signatures across various stages of HIV *versus* SIV infection, with an emphasis on the impact of microbiome-based therapies in restoring gut mucosal immunity as well as their translational potential to supplement current HIV cure efforts.

## Introduction

**FIGURE 1:**
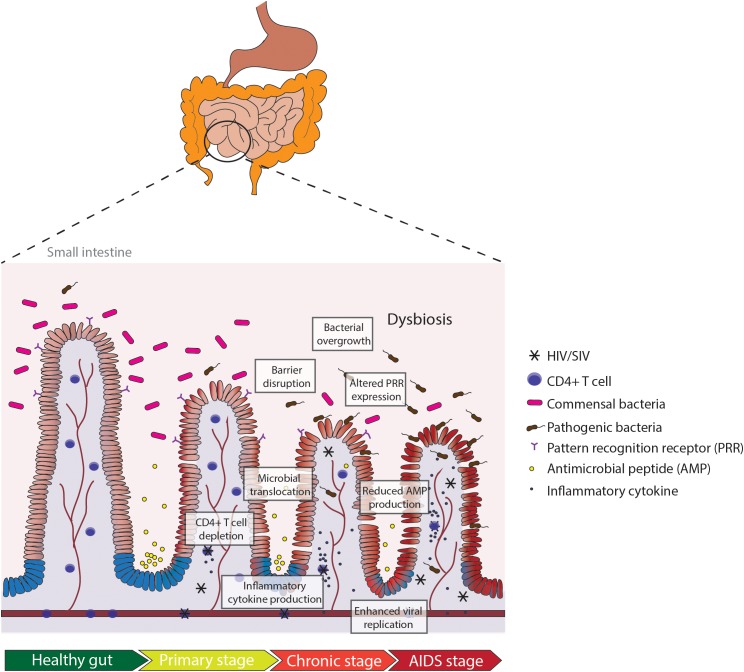
Pathologic manifestations of the small intestine in HIV or SIV infection in the gut. Depletion of CD4^+^ T cells occurs in early HIV infection (primary stage). Production of inflammatory cytokines in the primary stage leads to intestinal barrier disruption and microbial translocation, exacerbating the inflammatory milieu and increasing viral replication. Dysregulation in mucosal immunity and reduced anti-microbial peptide (AMP) production leads to bacterial overgrowth and altered pattern recognition receptor (PRR) expression in chronic HIV infection. Persistent gut inflammation in response to the cascade of mucosal events leads to enhanced viral replication and severe depletion of CD4^+^ T cells in the AIDS stage.

The human gastrointestinal (GI) tract plays a unique role in structural and immunological protection against exposure to the outside environment. The mucosal surface of the GI tract comes in contact not only with food and environmental antigens, but also with microorganisms such as bacteria, fungi, and viruses. For this reason, the GI tract is an important immunological site for maintaining the delicate balance between tolerance and reactivity (MacDonald and Monteleone, 2005; Lathrop et al., 2011). The coordination of innate and adaptive immune responses in gut-associated lymphoid tissues (GALT) is critical for rapid and long-term protection against pathogens (MacDonald and Monteleone, 2005). Evidence shows that both HIV-1 (HIV) and its non-human primate counterpart SIV target GALT as major sites of viral transmission, replication and seeding, and CD4^+^ T cell depletion, as depicted in Figure 1 (Heise et al., 1993, 1994; Lim et al., 1993; Veazey et al., 1998). In fact, the depletion of CD4^+^ T cells is more rapid in the gut during HIV and SIV infection in comparison to depletion in the peripheral blood (Veazey et al., 1998; Mehandru et al., 2007). Various host (IL-1β-induced pyroptosis, Fas-ligand, and TNF-α) and viral factors (HIV-1 Tat, Nef) have been proposed to contribute to massive CD4^+^ T cell loss in both HIV and SIV infection (Li et al., 2008; Doitsh et al., 2014). The loss of CD4^+^ T cells persists through the chronic stage of infection, in which most of the cell death seems to be driven by bystander killing of CD4^+^ T cells that were not actively infected (Finkel et al., 1995). Altered T cell homeostasis in the gut, particularly of CD4^+^ Th17 cells, coincides with disruption of intestinal barrier function, in which the tightly opposed enterocytes become “leaky” and lose their adherence to adjacent cells (Dandekar et al., 2010). Reports of increased and dysregulated IL-10 production in SIV and HIV infection have also been linked to intestinal permeability and inflammatory signatures (Pan et al., 2014; de Medeiros et al., 2016). Intestinal barrier disruption leads to translocation of microbial products from the lumen to systemic circulation, traveling to far-reaching organs such as the liver and brain and inducing persistent immune activation (Brenchley et al., 2006; Estes et al., 2010).

Like many diseases associated with GI inflammation, HIV alters the composition of gut microbes shortly after infection, which contributes to both intestinal barrier damage and altered mucosal immune responses (Gori et al., 2008; Ellis et al., 2011). Significant shifts in gut microflora, or dysbiosis, as a result of HIV infection, remain unresolved despite initiation of anti-retroviral therapy (ART). ART effectively suppresses HIV replication, but it cannot eradicate HIV reservoirs, or completely restore CD4^+^ T cells both in the gut and periphery in HIV positive individuals. Further, restoration of CD4^+^ T cells in the gut is delayed compared that in the peripheral blood (Stein et al., 1997; Guadalupe et al., 2006). Notably, initiation of ART fails to reduce chronic immune activation and markers of microbial translocation such as LPS and sCD14 (Marchetti et al., 2008; Wallet et al., 2010). Understanding the host-microbe interaction at the gut mucosal interface is critical in mitigating microbial translocation and immune activation in HIV-infected individuals under suppressive ART. As such, much effort has been made in recent years to investigate the significance of an altered gut microbiome during SIV or HIV infection. Clear changes in gut microbiota composition have been reported between HIV-infected and uninfected individuals, but evidence of gut dysbiosis has not been consistent in SIV-infected macaques (Supplementary Table 1). This review will discuss HIV and SIV-induced microbiome alterations in a comparative approach and highlight the implications of gut mucosal immune recovery in the efforts for an HIV cure.

## Internal Defenses: Gut Microbiota Is Associated With Host Immune Responses in HIV and SIV Infection

Understanding the interplay between gut microbiota and viral infection are paramount to support mucosal recovery for a healthy gut during ART. Investigation into (1) how SIV/HIV infection can shape gut microbiota and (2) the extent to which gut microbiota can influence immune responses to viral infection are both critical in the development of HIV cure strategies (Dillon et al., 2016). Shifts in microbiome composition in HIV have been attributed to several underlying causes, including a loss of appropriate host innate and adaptive immune responses that keep resident gut microbes at bay. HIV induces massive depletion of Th17 cells, a subset of CD4^+^ T cells that control intestinal bacteria and limit microbial translocation (Brenchley et al., 2008). Loss of Th17 cells coincides with disruption of intestinal barrier function, in which the tightly opposed enterocytes become “leaky” and lose their adherence to adjacent cells (Dandekar et al., 2010). The loss of tight junctions in intestinal barriers allows translocation of microbial products from the lumen to systemic circulation, inducing persistent immune activation in patients under ART (Brenchley et al., 2006; Estes et al., 2010). In addition, the imbalance in Th17 cells coincides with increases in Treg cells during HIV infection, leading to inappropriate tolerance of microbes and suppression of viral clearance (Li et al., 2011). Microbial products that cross the intestinal barrier interfere with pattern recognition receptor (PRR) expression in the gut mucosa, inhibiting appropriate cellular responses to combat viral infection and perpetuating gut inflammation (Elinav et al., 2011; Glavan et al., 2016). Accordingly, studies have reported that both TLR4-dependent LPS and TLR2-dependent peptidoglycan recognition induce CD4+ T cell activation (Brenchley et al., 2006; Marchetti et al., 2013; Neff et al., 2018). Host recognition of microbial antigens *via* toll-like receptors (TLRs) can augment viral infectivity, highlighting the importance of both bacterial and viral control in the gut mucosa during HIV infection (Kuss et al., 2011).

Human Immunodeficiency Virus or SIV infection-induced gut microbiota changes are linked to the complex and dynamic selection of potentially pathogenic bacteria, expansion of the enteric virome, and disruption in gut mycobiota, all of which can induce inflammation in the gut mucosa. First, fecal bacterial communities isolated from HIV positive individuals have been shown to increase pro-inflammatory production of TNF-α and IL-6 in monocytes (Neff et al., 2018). Bacteria belonging to the Proteobacteria phylum enhanced indoleamine 2,3-dioxygenase (IDO) activity and increased metabolism of tryptophan into kynurenine derivatives, corresponding with an imbalance of Th17/Treg during chronic HIV infection and in HIV-infected subjects under ART (Jenabian et al., 2013; Vujkovic-Cvijin et al., 2013). Bacterial-induced IDO expression can pervade the gut-brain axis, potentially contributing to depression, dementia, and neurocognitive dysfunction in HIV infection (Sardar and Reynolds, 1995; Ciesla and Roberts, 2001). Second, it is also clear that both viral and bacterial contribute to HIV and SIV-induced enteropathy in a very interconnected process. An expansion in the gut virome, including adenoviruses, picornaviruses, parvoviruses, and caliciviruses, occurs in SIV infection and is associated with levels of serum LPS-binding protein, suggesting that viral communities may be involved in regulation of commensal gut microbes and host immunity (Handley et al., 2012, 2016). Lastly, gut microbiota changes are highly influenced by fungi that share similar niches on the mucosal surface. Treatment of mice with antifungal drugs exacerbates DSS-induced colitis and leads to intestinal dysbiosis of bacterial populations, including decreased relative abundances of *Bacteroidetes*, *Clostridium*, and *Lactobacillus* spp. (Wheeler et al., 2016). Altogether, these findings imply that more knowledge is needed about the causes and consequences of gut dysbiosis in conjunction with viruses and fungi during HIV infection to improve therapies for immune recovery in the gut.

## A Comparison of Gut Microbiota Signatures in HIV *Versus* SIV Infection

### Early HIV or SIV Infection

Investigation in the early stages of HIV and SIV infection revealed that gut inflammation and loss of CD4^+^ T cells occur rapidly after viral exposure (Veazey et al., 1998; Guadalupe et al., 2003). While challenging studies of early HIV infection, due to detection and recruitment of recently exposed individuals, a study of 57 early stage HIV-infected, ART-naïve patients reported that 50% of individuals had increased gut inflammation and a breakdown of the intestinal barrier as indicated by fecal calprotectin (Gori et al., 2008). Plasma levels of Intestinal-fatty acid binding protein (I-FABP) and soluble suppression of tumorigenicity 2 (sST-2) were elevated in a cohort of 48 early stage HIV-infected patients, confirming the early onset of enterocyte damage and gut inflammation in HIV infection (Jenabian et al., 2015). In HIV-infected patients, inflammatory markers were concurrent with an altered fecal microbiome composition featuring increased abundances of *Pseudomonas aeruginosa* and *Candida albicans* and decreased abundances of *Lactobacilli* and Bifidobacteria (Gori et al., 2008). A reduction in *Lactobacillales* in early HIV infection was associated with increased microbial translocation, higher viral loads, and lower CD4^+^ T cell percentages (Perez-Santiago et al., 2013). Nevertheless, many early infection studies have been aimed largely at the SIV model in part because the early signs of gut inflammation are analogous to that in HIV infection. At 2.5 days post SIV-infection, Hirao et al. (2014) reported heightened IL-1β signaling and disruption of intestinal barriers prior to CD4^+^ T cell loss. Furthermore, induction of interferon (IFN)-α, IFN-γ, TNF-α, IL-8, IL-12, IL-17, IL-22, and IL-23 were elevated in the jejunal compartments of early SIV-infected rhesus macaques (Glavan et al., 2016). The alterations in gut microbiome composition have been relatively consistent between early HIV and SIV infection. In a study of 14 acutely SIV-infected rhesus macaques, fecal microbial analysis revealed a significant decrease in alpha (number of species and species evenness) and beta diversity (differences in taxonomic abundance profiles) with depletion of multiple *Lactobacillus* and *Streptococcus* spp (Vujkovic-Cvijin et al., 2015). Furthermore, jejunal microbiota analysis from 4 acutely SIV-infected macaques revealed an increase in *Pasteurellaceae* and trend of decrease in *Streptococcus* (Glavan et al., 2016). However, one study comparing stool samples from 9 acutely SIV-infected macaques with eight healthy controls revealed no strong clustering of bacterial communities by SIV status (McKenna et al., 2008). Bacterial clustering was observed when grouped by presence of colitis, highlighting the connection between gut inflammation and microbiome composition. Collectively, these findings support that the presence of gut inflammation in both early SIV and HIV infection can result in an altered microbiome composition.

### Chronic HIV or SIV Infection

Chronic HIV and SIV infection are both characterized by severe CD4^+^ T cell depletion, high levels of microbial translocation, and persistent immune activation. In a study of 20 chronic, ART-naïve HIV-positive men, fecal microbiota analysis revealed lower alpha diversity, lower abundances of Firmicutes, and increased abundances of Fusobacteria when compared to 20 uninfected controls (McHardy et al., 2013). Similarly, another study of 11 individuals in the chronic stage of HIV infection also reported significant differences in fecal microbiota when compared to healthy controls, highlighting an increase in alpha diversity and relative abundances of Prevotellaceae, Erysipelotrichaceae, and Veillonellaceae (Lozupone et al., 2013). In a span of 3 years, an upsurge of interest in studying the fecal microbiome in ART-naïve HIV-infected individuals revealed increased abundances of Proteobacteria (Vujkovic-Cvijin et al., 2013; Dillon et al., 2014; Ling et al., 2016) and *Prevotella* (Lozupone et al., 2013; Dillon et al., 2014; Ling et al., 2016) with decreased abundances of *Bacteroidetes* (Lozupone et al., 2013; Vujkovic-Cvijin et al., 2013; Dillon et al., 2014; Ling et al., 2016), Firmicutes (McHardy et al., 2013; Dillon et al., 2014), and *Erysipelotrichaceae* (Lozupone et al., 2013; Vujkovic-Cvijin et al., 2013) were associated with chronic HIV infection. Later studies comparing the impact of sexual preference on gut microbiota composition revealed that the increased abundance of *Prevotella* found in HIV infection was attributed to men who have sex with men (MSM) independent of HIV status (Noguera-Julian et al., 2016). Subtle changes in the fecal microbiome composition vary at higher taxonomic classifications. Studies report depletion in *Lachnospira*, *Coprococcus*, *Roseburia* and *Alistipes* and enrichment in *Peptostreptococcus*, *Anarococcus*, *Porphyromonas*, *Fusobacterium*, Veillonellaceae, Ruminococcaceae, and Desulfovibrionaceae associated with HIV infection (Lozupone et al., 2013; McHardy et al., 2013).

The magnitude of changes in gut microbiota composition during the chronic stage of SIV infection in non-human primates, however, have not corresponded well with that of chronic HIV infection in patients. Many similarities were found in the fecal microbiota of healthy humans and macaques, but unlike findings in HIV infection, there was no strong clustering of bacterial communities associated with SIV infection (McKenna et al., 2008; Klatt et al., 2013). Fecal microbial analysis from 8 ART-naïve, chronic SIV-infected rhesus macaques noted a gradual decrease in *Lactobacillus* over time, but no other significant changes in microbiome composition compared to that from 3 uninfected controls (Klase et al., 2015). Similarly, a study involving 22 SIV-infected rhesus macaques reported no SIV-associated differences in bacterial richness, evenness, nor diversity when compared to 22 uninfected controls (Handley et al., 2012). Microbial analysis from colon biopsies of 4 chronic SIV-infected macaques revealed subtle decreases in abundance of Firmicutes and increases in *Mycooplasmataceae*, suggesting the possibility of minor distinctions in mucosal-adherent bacterial populations (Glavan et al., 2016). Investigation into the fecal microbiome chimpanzees naturally infected with SIVcpz, the ancestral strain that transmitted to humans as HIV-1, proved to be distinct from findings in the rhesus macaque. An increase in relative abundances of *Sarcina*, *Staphylococcus*, *Selenococcus*, and *Tetragenococcus* were observed in fecal samples from 6 chronic SIV-infected chimpanzees (Moeller et al., 2013). Collectively, these findings show that the gut microbiome alterations due to chronic SIV infection in non-human primates are variable and display quite different profiles than that in chronic HIV-infected individuals. The exception remains in the SIVcpz model, and the reason for this discrepancy is unclear. Speculations about environmental influences, including primate housing conditions, diet, and length of infection on gut microbial compositions may limit comparisons across studies. The variability of environmental conditions is not controlled in human studies and natural non-human primate infections compared with the widely used experimental AIDS model of rhesus macaques, which highly influence outcomes of analysis in gut microbial communities (Conlon and Bird, 2014; Coad et al., 2017). Furthermore, the rate of disease progression for chronic pathogenic SIV infection is defined within several months in comparison to several years in HIV infection which may lead to inherent differences in gut microbial composition. While differences in gut microbiota signatures are seen in chronic infection, the pathologic manifestations of HIV and SIV infection in the gut retain many similarities, laying the groundwork for seminal research in early establishment and latency of viral reservoirs, microbial translocation, and immune activation (Chahroudi and Silvestri, 2016; Garcia-Tellez et al., 2016; Kumar et al., 2016; Huot et al., 2018).

### Limitations to Current Gut Microbiome Studies in HIV or SIV Infection

Microbiome-related changes in the context of HIV, SIV, and other chronic inflammatory diseases have been a constant challenge to fully elucidate due to a variety of confounding factors. First, there lacks a consistent method for fecal sample collection. Samples obtained from stool, colon contents, and colon biopsies reveal different microbial compositions that may influence detection of bacterial species across studies (McKenna et al., 2008). Secondly, the methods to preserve collected samples are not clearly described in these studies and may strongly influence the analysis of bacterial communities (Horng et al., 2018). Host factors beyond HIV and SIV status can impact the gut microbiome and create disparity among studies in different populations, including exposure to subclinical viruses and environmental conditions (Santos Rocha et al., 2018). Unlike non-human primates whose daily housing condition and diets are similar, the diet habits of HIV-infected individuals vary among persons, genders, areas, and seasons, which may account for differences in microbiota. One such example is the enrichment of environmentally derived bacterial genera in the gorilla gut microbiota due to their specialized leaf-based diet and capacity to digest complex plant polysaccharides (Moeller et al., 2015). Our findings highlight a need to consider host, environmental, and viral factors in gut microbiome analyses between SIV and HIV infection. While current studies have established correlations between changes in gut microbiota and HIV disease progression, the challenge now lies in moving beyond correlation analyses to begin addressing causation. More resources are needed to encourage mechanistic studies and enable identification of causal microbes in HIV disease pathogenesis.

## Impacts of Art Treatment on Gut Microbiota Composition

Administration of ART can increase CD4^+^ T cell counts and reduce viral loads in plasma, but it fails to eradicate HIV reservoirs, normalize the composition of the gut microbiome, and resolve gut inflammation (Dinh et al., 2015; Nowak et al., 2015). One study involving 6 chronic HIV individuals on a short course of ART revealed that short-term administration of ART does not restore fecal microbiota composition to that of healthy controls (Lozupone et al., 2013). In contrast, long-term use of ART during chronic HIV infection may partially restore gut microbiota, but it does not completely normalize the microbial composition to that of healthy individuals (Lozupone et al., 2013; McHardy et al., 2013; Ling et al., 2016). Similar findings in SIV infection revealed that treatment using ART resulted in an initial decrease in abundance of Bacteroidetes and Firmicutes and increase in Proteobacteria, which normalized to levels in untreated SIV-infected controls after 2 weeks (Klase et al., 2015). In comparison to HIV-negative individuals, chronic ART-treated HIV individuals still exhibited reduced alpha diversity (Mutlu et al., 2014; Nowak et al., 2015), increased abundances of *Succinivibrio* (Vázquez-Castellanos et al., 2014) and *Enterobacteriaceae* (Mutlu et al., 2014; Dinh et al., 2015), and decreased abundances of *Bacteroidetes* (Vázquez-Castellanos et al., 2014; Nowak et al., 2017), *Alistipes* (Dinh et al., 2015), *Erysipelotrichaceae* (Dinh et al., 2015), similar to that of chronic, ART-naïve HIV individuals. Two recent studies have examined the effects of different ART combinations on the gut microbiome. Pinto-Cardoso et al. (2017) showed that long-term use of ART reduced relative abundances of *F. prausnitzii* and Roseburia, potential butyrate producers that help maintain healthy gut homeostasis. In particular, a protease inhibitor (PI)-based ART regimen increased endothelial damage and exhibited higher levels of sCD14 in plasma when compared to both the non-nucleoside reverse transcriptase inhibitor (NNRTI)-based ART and HIV-uninfected controls. The detrimental effects of PIs were consistent with findings from Villanueva-Millan et al. (2017) highlighting the reduction of beneficial butyrate-producing bacteria in HIV infection. A comparison of PI, NNRTI, and integrase-strand transfer inhibitor (INSTI)-based ART regiments revealed that INSTI use was associated with the lowest levels of systemic inflammation and minor changes in fecal microbial composition compared to healthy controls (Villanueva-Millan et al., 2017). The significance of the gut microbiome has been increasingly recognized in development of ART for HIV infection, but much is still unknown about differential effects of drugs on commensal microbes critical for gut health. These findings highlight the need to better understand the pharmacokinetics of oral ART drugs and their interactions with gut microbial communities to achieve complete mucosal recovery.

**FIGURE 2:**
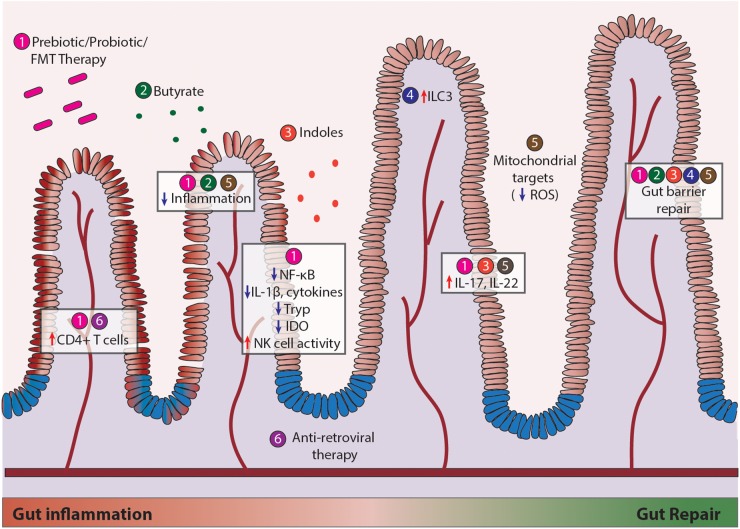
Current therapeutic modalities to repair mucosal damage during HIV and SIV infection. Strategies include (1) recovery of gut microbial communities, (2) prebiotic stimulation using butyrate, (3) addition of bacterial metabolites such as indoles, (4) enhancing host immune cell function through innate lymphoid cells (ILC), (5) combating cellular metabolism through mitochondrial targets such as ROS, and (6) anti-retroviral therapy (ART). Efforts made in regard to each strategy (Heise et al., 1993, 1994; Lim et al., 1993; Veazey et al., 1998; MacDonald and Monteleone, 2005; Lathrop et al., 2011) have led to modest increases in CD4^+^ T cell numbers, reduced inflammation, increased NK cell activity, enhanced IL-17/IL-22 production, and gut epithelial barrier repair.

## Establishing a Stronghold: Restoring Gut Microbiota Composition in HIV/SIV Infection

Restoring the gut microbiome in HIV infection using probiotics and prebiotics have a multitude of implications on intestinal barrier functions, resistance to pathogenic colonization, and restoration of the Th17/Treg ratio for mucosal immune function (D’Angelo et al., 2017), as depicted in Figure 2. Probiotic supplementation in HIV infection has been shown to moderately improve CD4^+^ T cell counts and alleviate GI symptoms such as diarrhea and nausea (Cunningham-Rundles et al., 2011; Yang et al., 2014; Miller et al., 2016). In a randomized, double-blinded study of 32 HIV patients receiving ART with 15 receiving probiotics, 9 receiving placebo, and 8 controls, probiotic intervention reduced systemic inflammatory C-reactive protein levels and CD4^+^ T cell activation. However, these anti-inflammatory effects occurred independent of changes in microbial translocation (Stiksrud et al., 2015). Other studies have found that use of prebiotics or probiotics can reduce activation of circulating CD4^+^ T cells, decrease production of inflammatory TGF-β, IL10, IL-12, and IL-1β, and improve NK cell activity, highlighting the clinical potential to support immune reconstitution and facilitate provirus clearance (Gori et al., 2011; d’Ettorre et al., 2015; Falasca et al., 2015). Interestingly, outcomes of probiotic/prebiotic supplementation in the SIV infection support findings in HIV infection despite distinctions in SIV and HIV-induced changes in the gut microbiome. Supplementation of probiotics/prebiotics during ART resulted in increased GI tract function, better reconstitution and functionality of intestinal CD4^+^ T cells, and reduced fibrosis in colonic lymphoid follicles in SIV infected pigtail macaque model of AIDS (Klatt et al., 2013). Probiotics (VSL#3) given with IL-21 and ART increases *Lactobacillus* and *Bifidobacteria* spp, increase polyfunctional IL-17 expansion, and reduce microbial translocation and IDO expression in the gut (Ortiz et al., 2016). Probiotic administration for 1 month reversed the depletion of *Lactobacillus* and increased circulating kynurenine levels in chronic SIV infection (Klase et al., 2015; Vujkovic-Cvijin et al., 2015). Inoculation of commensal *Lactobacillus plantarum* in the intestinal loops led to a rapid anti-inflammatory response and epithelial tight junction repair by dampening SIV-infection-induced NF-κB/IL-1β signaling in gut of early SIV-infected macaques (Hirao et al., 2014). In addition, fecal microbiota transplantation in SIV-infected rhesus macaques enhanced frequencies of circulating Th17 and Th22 cells responsible for maintaining epithelial homeostasis (Hensley-McBain et al., 2016). These studies suggest that microbial-based therapies strongly support mucosal health and should be considered in conjunction with ART. Furthermore, restoring gut heath in combination with anti-latency strategies may have implications for current HIV cure efforts to fully eradicate or prevent viral infection. Care should be taken in consideration for selection of specific probiotic strains, as evidence suggests that probiotics are not all equally effective (Nazir et al., 2018). More research is warranted to elucidate the mechanism of how microbes interact with the gut in inflammatory conditions, evaluate the safety of probiotic use, and understand their potential in maintaining long-term health benefits. While observational microbiome studies provide useful information, the key to understanding their therapeutic potential lies in interventional studies at the species level.

## Alternative Strategies to Rescue Gut Mucosal Immunity

Insights into the beneficial outcomes of probiotic use has paved the way for development of more specific targets of gut mucosal immunity. Administration of Sevelamer to neutralize microbial LPS was utilized in SIV-infected macaque model, which dramatically reduced immune activation and slightly reduced viral replication (Kristoff et al., 2014). However, in ART-naïve HIV infected patients Sevelamer failed to decrease microbial translocation, inflammation or T-cell activation (Sandler et al., 2014), suggesting that LPS may not be the sole driver of disease progression in HIV-infected individuals and alternative strategies are needed to target mucosal immune system. One new potential avenue of research involves metabolites of bacterial origin that can influence host cellular functions. Bacterial-derived short chain fatty acids (SCFAs) such as butyrate and acetate have been shown to support epithelial barriers, have anti-inflammatory properties, and influence cellular metabolism (Koh et al., 2016; Byndloss et al., 2017). Bacterial-derived indole derivatives can activate aryl hydrocarbon receptors (AhR) to induce IL-22 production, decrease inflammatory cytokines, and prevent gut leakiness (Zelante et al., 2013; Lamas et al., 2016; Choi et al., 2018). Research efforts to improve gut mucosal immunity have also been focused on fine-tuning host cellular signaling pathways that are altered by HIV infection. Modification of innate lymphoid cell 3 populations can regulate commensal bacteria-specific CD4^+^ T cells (Hepworth et al., 2015). Permissiveness of T-cells to HIV infection may be partially controlled by mTOR activity (Planas et al., 2017). Progression of gut inflammation in HIV infection may be corrected through inhibition of NF-κB in the host (Hirao et al., 2014; Vlantis et al., 2016). Lastly, targeting cellular mitochondrial metabolism and antioxidant potential may be of importance to maintain epithelial barrier integrity and homeostasis in the gut (Wang et al., 2014; Han et al., 2017). Gut inflammation generates oxygen and nitrogen radicals that may further contribute to dysbiosis by allowing outgrowth of anaerobes (Winter et al., 2013). Restriction of nitrogen availability to gut microbiota through modulation of diet helped shaped microbial communities and promote metabolic health in mice (Holmes et al., 2017). The mechanisms of how metabolic function in the gut is altered in HIV and SIV infection and how this can be targeted therapeutically to promote both gut and brain health is under active investigation.

## Concluding Remarks and Future Perspectives

The mechanisms of how the gut microbiota is involved in the host inflammatory processes that influence HIV replication and disease progression are just beginning to be elucidated. Studies in the SIV model have been an invaluable tool to study gut inflammation associated with HIV despite differences observed in gut microbiota compositions. Studies in non-human primates have contributed greatly to our working knowledge of viral transmission, gut-associated inflammation, early immune responses, and prevention and treatment strategies. While development of novel technologies characterized key bacteria that affect mucosal immunity, more research is needed to identify the mechanisms influencing gut health and long-term impacts of microbiome-based therapies. For example, production of SCFA by gut microbiota were altered during HIV infection, which may contribute to gut mucosal damage (Qing et al., 2018). Interestingly, SCFA is essential for a newly discovered epigenetic modification – histone crotonylation (Fellows et al., 2018). We recently discovered that histone crotonylation involved in HIV replication and decrotonylation of histone tails at HIV long terminal repeats (LTR) may be linked to the establishment of HIV latency (Jiang et al., 2018). These recent findings indicate that we have yet to unravel signaling pathways critical for HIV disease progression and gut mucosal damage, which require the development of powerful computation tools, such as bacterial transcriptome and epigenetic analyses in combination with host genetic and metabolomics analyses. These cohesive studies will help us develop novel strategies to restore gut health and eliminate HIV reservoirs in patients. Harnessing the SIV model to study host-microbial interactions at the molecular level could provide great insight in restoring gut mucosal immunity in HIV infection. Concurrent strategies to restore mucosal damage, suppress viral replication, and reduce reservoir size in patients are key to the success of HIV cure efforts.

## Author Contributions

GJ initiated the concept of this manuscript. KC and GJ revised multiple versions of this review and approved the manuscript.

## Conflict of Interest Statement

The authors declare that the research was conducted in the absence of any commercial or financial relationships that could be construed as a potential conflict of interest.
